# The effects of attention in auditory–visual integration revealed by time-varying networks

**DOI:** 10.3389/fnins.2023.1235480

**Published:** 2023-08-02

**Authors:** Yuhao Jiang, Rui Qiao, Yupan Shi, Yi Tang, Zhengjun Hou, Yin Tian

**Affiliations:** ^1^ Institute for Advanced Sciences, Chongqing University of Posts and Telecommunications, Chongqing, China; ^2^ Guangyang Bay Laboratory, Chongqing Institute for Brain and Intelligence, Chongqing, China; ^3^ Central Nervous System Drug Key Laboratory of Sichuan Province, Luzhou, China

**Keywords:** auditory–visual integration, attention, time-varying network connectivity, fMRI, EEG

## Abstract

Attention and audiovisual integration are crucial subjects in the field of brain information processing. A large number of previous studies have sought to determine the relationship between them through specific experiments, but failed to reach a unified conclusion. The reported studies explored the relationship through the frameworks of early, late, and parallel integration, though network analysis has been employed sparingly. In this study, we employed time-varying network analysis, which offers a comprehensive and dynamic insight into cognitive processing, to explore the relationship between attention and auditory-visual integration. The combination of high spatial resolution functional magnetic resonance imaging (fMRI) and high temporal resolution electroencephalography (EEG) was used. Firstly, a generalized linear model (GLM) was employed to find the task-related fMRI activations, which was selected as regions of interesting (ROIs) for nodes of time-varying network. Then the electrical activity of the auditory-visual cortex was estimated via the normalized minimum norm estimation (MNE) source localization method. Finally, the time-varying network was constructed using the adaptive directed transfer function (ADTF) technology. Notably, Task-related fMRI activations were mainly observed in the bilateral temporoparietal junction (TPJ), superior temporal gyrus (STG), primary visual and auditory areas. And the time-varying network analysis revealed that V1/A1↔STG occurred before TPJ↔STG. Therefore, the results supported the theory that auditory-visual integration occurred before attention, aligning with the early integration framework.

## 1. Introduction

Individuals are constantly exposed to a plethora of sensory information that they unconsciously integrate in order to comprehend their environment. Visual and auditory information constitutes the majority (over 90%) of the information that is perceived (Treichler, 1967; Ristic and Capozzi, 2023). Auditory–visual integration occurs when auditory and visual stimuli coincide temporally and spatially, and when two stimuli are presented within a close time interval and similar spatial arrangement (Stein and Meredith, 1990; Frassinetti et al., 2002; Stevenson et al., 2012; Spence, 2013; Tang et al., 2016; Ľuboš et al., 2021). Attention plays a crucial role in selectively processing external information and improving information processing performance through focusing on target locations (Posner and Rothbart, 2006; Zhang T. et al., 2022). Attention is instrumental in processing dynamic stimuli efficiently and enhancing perception, as it directs limited cognitive resources toward information relevant to the current task (Tian et al., 2014; Li et al., 2015). In addition, the researches on the attention mechanism may help to improve deep neural networks for visual processing tasks (Zhang et al., 2019; Wang et al., 2020).

There is ongoing debate regarding the role of attention in multisensory integration, particularly in the case of auditory–visual integration. Three mainstream theories about the relationship between auditory–visual integration and attention were proposed in previous studies (Koelewijn et al., 2010; Xu et al., 2020). The first, the early integration framework, asserts that integration occurs prior to attention and can even drive it (Vroomen et al., 2001; Rachel et al., 2022). Evidence for this is seen in the “pip-pop effect,” where the addition of auditory stimulation to a visual search task led to faster results (Erik et al., 2008). Then non-spatial auditory stimulation was added to the spatial visual experiment. The second theory, the late integration framework, demonstrates that multisensory integration appears behind attention. In other words, two unimodal (i.e., auditory and visual) events are attended to separately before they are integrated. This model indicates that attention is necessary for multisensory integration (Laura et al., 2005; Sébastien et al., 2022). A later study used a cross-modal attention preference task to prove that cross-modal interactions are influenced by attention (Romei et al., 2013; Wen et al., 2021). Furthermore, late integration suggests that late cross-modal effects are mediated by attentional mechanisms. The third theory is the parallel integration framework; here, the stage at which multisensory integration takes place is uncertain. Multisensory integration can be early or late, and it depends on experimental or external conditions (Calvert and Thesen, 2004; Sébastien et al., 2022). Some studies extended the seminal methods of the parallel integration framework (Talsma et al., 2010; Stoep et al., 2015). This may produce different results as a result of several factors, including task type (detection or identification), stimulus properties (simple or complex), and attention resources (exogenous or endogenous).

In the study of the relationship between attention and auditory–visual integration, various methods have been employed. Early research utilized behavioral data and discovered that an auditory stimulus influences the reaction time (RT) of a synchronous or nearly synchronous visual stimulus (Mcdonald et al., 2000; Shams et al., 2000; Laura et al., 2005; Zhang X. et al., 2022) and the reverse is also true (Platt and Warren, 1972; Bertelson, 1999). These results indicate that a simultaneous or near-simultaneous bimodal stimulus reduces stimulation uncertainty (Calvert et al., 2000), potentially supporting the early integration framework or enhancing stimulation response for the late framework (Stein et al., 1989; Zhang et al., 2021). However, external factors, such as the state of the experimental subjects, may be overlooked.

With the advancement of brain imaging technology, increasing numbers of researchers have turned to brain imaging to investigate the relationship between attention and auditory–visual integration. By utilizing an event-related potential component (ERP) of an auditory–visual streaming design and a rapid serial visual presentation paradigm, they explored the interactions between multisensory integration and attention (Durk and Woldorff, 2005; Kang-jia and Xu, 2022). The results indicated that activity associated with multisensory integration processes is heightened when they are attended to, suggesting that attention plays a critical role in auditory–visual integration and aligning with the late integration criteria. The improvement of the spatial resolution of scalp EEG has long been a subject of interest for researchers.

Studies using functional magnetic resonance imaging (fMRI) with high spatial resolution have reported the accurate location of many areas involved in auditory–visual integration and attention; these mainly include the prefrontal, parietal, and temporal cortices (Calvert et al., 2001; Macaluso et al., 2004; Tedersälejärvi et al., 2005; Noesselt et al., 2007; Cappe et al., 2010; Chen et al., 2015).The superior temporal gyrus (STG) and sulcus (STS) both participate in speech auditory–visual integration (Klemen and Chambers, 2012; Rupp et al., 2022) and non-speech auditory–visual stimuli (Yan et al., 2015). In the past, STG was considered an area of pure sound input (Mesgarani et al., 2014). The temporoparietal junction (TPJ), which is close to the STG, is an important area of the ventral attention network (VAN) that is located mostly in the right hemisphere, and is recruited at the moment a behaviorally relevant stimulus is detected (Corbetta et al., 2008; Tian et al., 2014; Branden et al., 2022). The TPJ is activated during detection of salient stimuli in a sensory environment for a visual (Corbetta et al., 2002, 2008), auditory (Alho et al., 2015), and auditory–visual task (Mastroberardino et al., 2015). However, as many studies have mentioned, it is difficult to determine accurately the timing characteristics when using fMRI with poor temporal resolution.

For the reason that EEG and fMRI are two prominent noninvasive functional neuroimaging modalities, and they demonstrate highly complementary attributes, there has been a considerable drive toward integrating these modalities in a multimodal manner (Abreu et al., 2018). The combination of scalp EEG’s exceptional temporal resolution and fMRI’s remarkable spatial resolution enables a more comprehensive exploration of brain activity, surpassing the limitations inherent to individual techniques (Bullock et al., 2021). Previous investigations have examined the functional aspects of the brain in various pathological conditions, such as schizophrenia (Baenninger et al., 2016; Ford et al., 2016). Multiple researchers have employed combination of EEG and fMRI to explore cognitive mechanisms (Jorge et al., 2014; Shams et al., 2015; Wang et al., 2018). Some other studies have investigated brain dynamics in relation to complex cognitive processes like decision-making and the onset of sleep (Bagshaw et al., 2017; Pisauro et al., 2017; Hsiao et al., 2018; Muraskin et al., 2018). In this study, we used these two neuroimaging technologies to investigate the appearance order of auditory–visual integration and attention. Previous studies have tended to apply a specific experimental paradigm to investigate this relationship, but few have used network analyses to resolve this conundrum. We employed time-varying network analysis based on the adaptive directed transfer function (ADTF) method to uncover dynamic information processing. This method can uncover the dynamic information processing with a multivariate adaptive autoregressive mode (Li et al., 2016; Tian et al., 2018b; Nazir et al., 2020). This approach may offer new insights into the temporal order of multisensory integration and attention in a stimulated EEG network.

## 2. Materials and methods

### 2.1. Participants

The data for this study was obtained through separate EEG and fMRI recordings, conducted on 15 right-handed, healthy adult males (mean ± standard deviation (SD) = 21.4 ± 2.8 years). Participants provided informed consent and were free from visual or auditory impairments and any mental health conditions. Upon completion of the experiments, participants were compensated for their time. The study was approved by the Ethics Committee of the University of Electronic Science and Technology of China.

### 2.2. Experimental design

**Figure 1:**
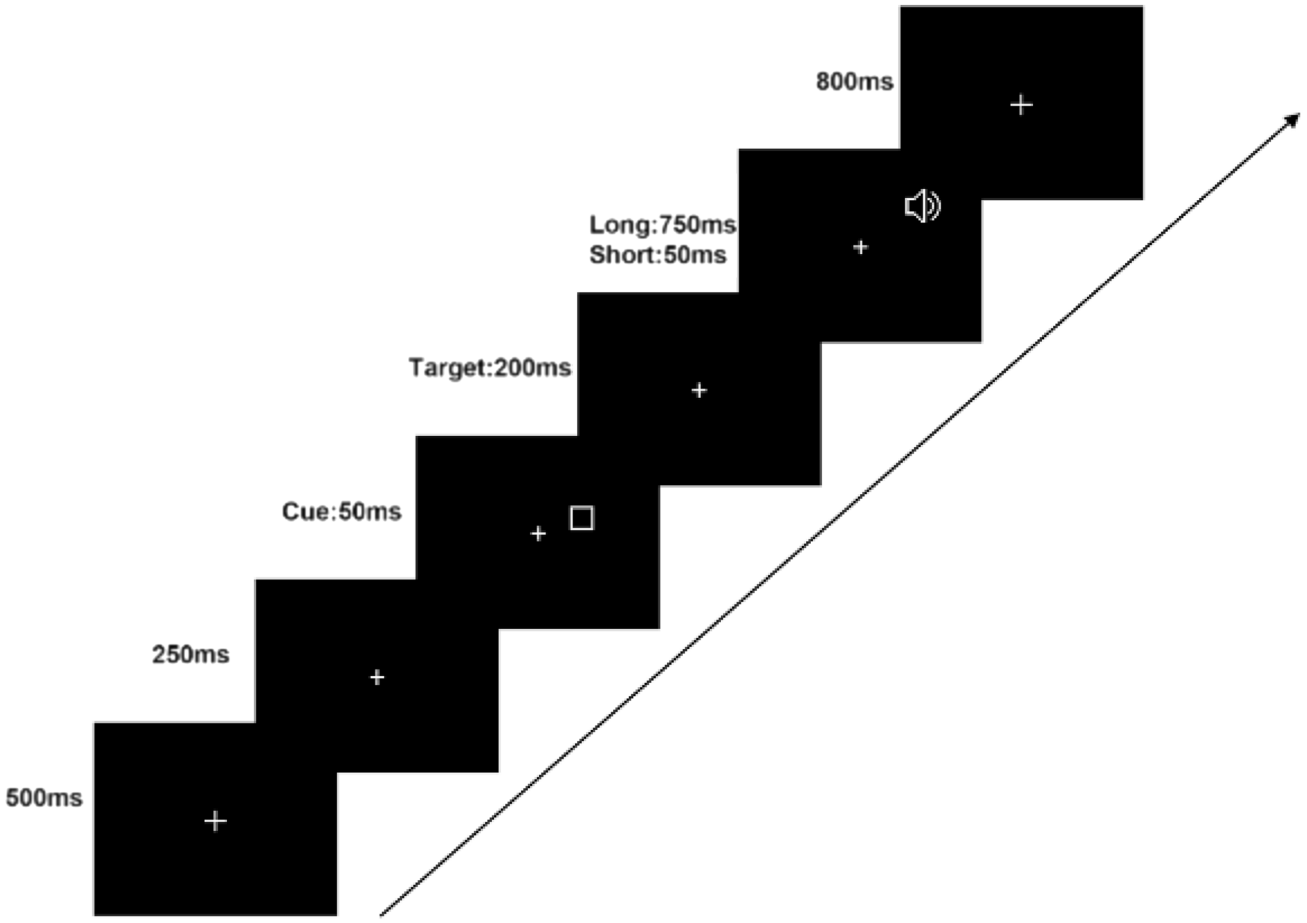
Illustration of stimulus sequence in the experiment.

Throughout the experiment, a white fixation cross of dimensions ( 0.5°×0.5° ) was presented at the center of a black monitor. The visual stimuli consisted of rectangular boxes that randomly appeared in either the left or right visual field (LVF or RVF, respectively). The box was 2°×2° and its width was 0.2° . The boxes remained on the screen for 50 ms and were followed by an auditory stimulus, a 1,000 Hz pure tone that also randomly appeared in the left or right auditory field (LAF and RAF, respectively) after a 50 or 750 ms interval. Participants were instructed to respond by pressing the ‘Z’ key with their left hand if the tone appeared in the LAF, and the ‘/’ key with their right hand if it appeared in the RAF. Participants were required to react as soon as they heard the pure tone, which lasted for 200 ms. The fixation cross remained on the monitor for an additional 800 ms to ensure participants had sufficient time to respond correctly. The experimental procedure is illustrated in Figure 1.

### 2.3. Behavioral data and analysis

The behavioral data was obtained via EEG and fMRI. We analyzed RT using repeated measures analysis of variance (ANOVA) with the following factors: stimulus visual field (LVF vs. RVF), cue validity (valid vs. invalid), stimulus-onset asynchrony (SOA), and the interval between the cue and target stimulus (long vs. short). Data consistency was ensured by excluding RTs greater than 900 ms and less than 200 ms, as well as any instances of missed or incorrect key presses.

### 2.4. EEG and fMRI data recording

In the study, EEG and fMRI data were collected separately. We used a Geodesic Sensor Net (GSN) with 129-scalp electrodes located according to the International 10–20 system (Tucker, 1993) to record the EEG at a rate of 250 Hz. The Oz, Pz, CPz, Cz, FCz, and Fz electrodes were placed in the middle of the skull, and the remaining electrodes were distributed along both sides of the midline. The central top electrode (Cz) was used as the reference electrode and all electrodes had impedances lower than 40 kΩ (Tucker, 1993).

fMRI data was collected using the fast T2*-weighted gradient echo EPI sequence on a 3-T GE MRI scanner (TR = 2000 ms, TE = 30 ms, FOV = 24 cm × 24 cm, flip angle = 90°, matrix = 64 × 64, 30 slices) at the University of Electronic Science and Technology of China. This method obtained 198 volumes for each session. Because the machine at was unstable at the beginning of the data collection, we discarded the first five image volumes of each run for preprocessing.

### 2.5. The processing framework for time-varying networks

**Figure 2:**
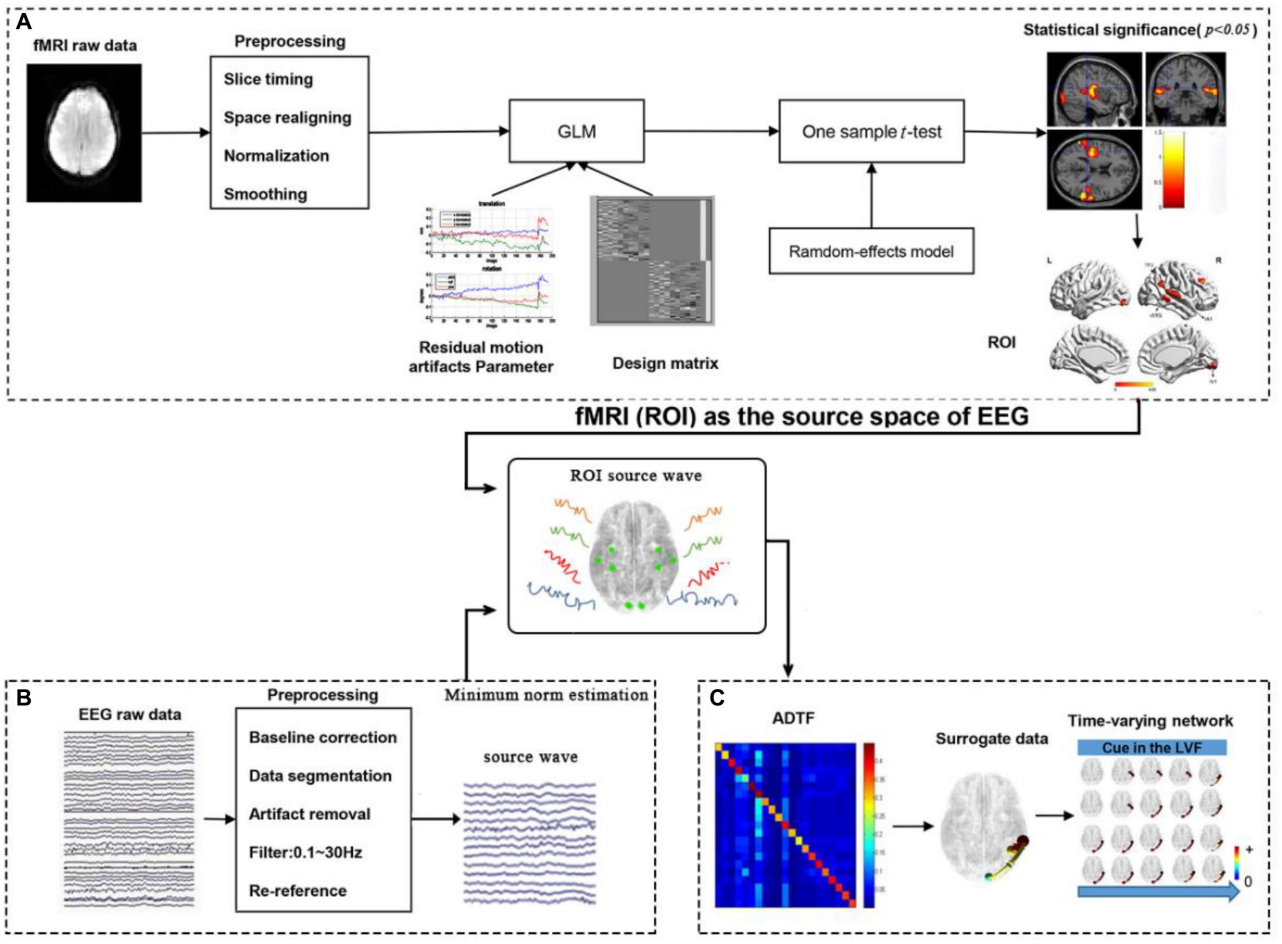
The processing framework for calculating time-varying networks. **(A)** fMRI processing; **(B)** EEG processing; **(C)** Time-varying network constructing.

The processing framework for calculating time-varying networks consisted of three stages, as illustrated in Figure 2.

1. ROI selection based on task-related fMRI activations, as shown in Figure 2A.

The fMRI data was preprocessed and constructed by a generalized linear model (GLM). The results of the GLM were then subjected to a statistical test. Reply on the statistical results, 4 activations for the left cue and 4 activations for the right cue in the fMRI experiment were selected as ROIs (nodes) in the cerebral cortex, providing relatively accurate MNI coordinates for the construction of the time-varying network in the following steps.

2. Source wave extraction (Figure 2B).

The EEG data was preprocessed, and the scalp electrical signals are mapped to the cerebral cortex by MNE source localization method. Then, the MNI coordinates provided by fMRI were converted to the corresponding positions of the head model and the corresponding time series of the cortical electrical signals are extracted.

3. Time-varying network construction (Figure 2C).

In the third stage, the time-varying network was constructed using the ADTF technology, based on the results from steps 1 and 2.

### 2.6. fMRI data processing

The remaining volumes underwent preprocessing using Statistical Parametric Mapping version 8 (SPM8) software. Four preprocessing pipelines were applied in this study. Firstly, slice timing correction was implemented to address temporal differences among the slices. Secondly, spatial realignment was performed to eliminate head movement, whereby all volumes were aligned with the first volume. Participants whose head movement exceeded 2 mm or 2 degrees were excluded (Bonte et al., 2014). Thirdly, normalization was carried out to standardize each participant’s original fMRI image to the standard Montreal Neurological Institute (MNI) space using EPI templates. Voxel resampling to 3 × 3 × 3 mm^3^ was performed to overcome head size inconsistencies. Lastly, spatial smoothing was implemented to ensure high signal-to-noise ratio (SNR) by smoothing the functional images with a Gaussian kernel of full width half maximum (FWHM) of 6 × 6 × 6 mm^3^.

After data preprocessing, the time series of all voxels underwent a high-pass filter at 1/128 Hz and were then analyzed with a general linear model (GLM; Friston et al., 1995) using SPM8 software. Temporal autocorrelation was modeled using a first-order autoregressive process. At the individual level, a multiple regression design matrix was constructed using the GLM, that included two experimental events based on the cue location (left visual field or right visual field). The two events were time-locked to the target of each trial by a canonical synthetic hemodynamic response function (HRF) and its temporal and dispersion derivatives. By including dispersion derivatives, the analysis accounted for variations in the duration of neural processes induced by the cue location. Nuisance covariates, such as realignment parameters, were included to account for residual motion artifacts. Parameter estimates were obtained for each voxel using weighted least-squares, which provided maximum likelihood estimators based on the temporal autocorrelation of the data (Wang et al., 2013).

In this study, to compute simple main effects for each participant, baseline contrasts were applied to the experimental conditions. Subsequently, the resulting individual contrast images were entered into a second-level one sample *t*-test using a random-effects model. In order to identify areas of significant activation, a threshold of *p* < 0.05 (false discovery rate [FDR] corrected) and a minimum cluster size of 10 voxels were utilized. These stringent criteria were employed to ensure robust and reliable identification of neural activation patterns.

### 2.7. EEG data preprocessing

The EEG data underwent five preprocessing steps. Firstly, the EEG epochs were set to a time range of −200 to 1,000 ms. Secondly, we used the average of 200 ms pre-stimulus data as a baseline to correct the epochs. Thirdly, we performed artifact rejection, excluding epochs contaminated by eye blinks, eye movements, amplifier clipping or muscle potentials that exceeded ±75 μv. Fourthly, we filtered the EEG recordings using a band-pass filter of 0.1-30 Hz. Finally, we re-referenced the data using the reference electrode standardization technique (REST) (Yao et al., 2005; Tian and Yao, 2013; Tian et al., 2018a). We excluded trials with incorrect behavioral responses and bad channel replacements, and averaged the ERPs from the stimulus onset time point based on the validity of the cue, visual field, and SOA length.

### 2.8. Minimum norm estimation

The volume conductor effect may lead to the generation of pseudo-connections during brain network construction using scalp brain electricity. And invasive methods for directly collecting brain electricity in the cerebral cortex are challenging to use. To overcome this problem, we employed source localization technology to transfer scalp brain electrical signal to the cortex, enabling estimation of cortical electrical signals (Tian et al., 2018a; Tian and Ma, 2020), and we converted 129 scalp electrodes into 19 electrodes covering the whole brain.

In this study, we used the normalized minimum norm estimation (MNE) source localization method to estimate the electrical activity of the auditory–visual cortex. Compared to other methods, the normalized MNE offers higher dipole positioning accuracy, especially in depth source analysis. Our head model consisted of a three-layer realistic representation of the cortex, skull, and scalp. The formula for MNE calculation is expressed as follows:


(1) φ(t)=ωx(t)

Where x(t) is the EEG collected by the scalp, φ(t) is the corresponding cortical EEG, and ω is the field matrix, which can be obtained from the following formula:


(2) $$\omega =C_{s}A^{T}{\left(AC_{s}A^{T}+\mu^{2}C_{n}\right)}^{-1}$$

where $C_{s}$ is the signal covariance, $C_{n}$ is the noisy covariance, and A is the transfer matrix obtained by the boundary element theory. μ is a regularization parameter and is obtained by the following formula:


(3) $$\mu =\frac{trace\left(AC_{s}A^{T}\right)}{trace\left(C_{n}\right)\times snr^{2}}$$

where snr is the signal to noise ratio.

### 2.9. Cortical time-varying network

The MNE source localization method was employed to transfer scalp electrical signals to the cerebral cortex. Next, MNI coordinates obtained from fMRI were mapped to the corresponding positions on the head model, and the cortical electrical signal time series at these positions were extracted. Subsequently, we designated these positions as nodes of the network and constructed the time-varying network using the relationship between these time series as the network edges.

To calculate the ADTF, we computed the multivariate adaptive autoregressive (MVAAR) model for all conditions. The model was normalized and expressed by following equation:


(4) $$X\left(t\right)=\overset{p}{\underset{j}{\sum }}\omega \left(j,t\right)X\left(t-j\right)+\eta \left(t\right)$$

where X(t) is the EEG data vector over the entire time window, ω(j,t) is the coefficient matrix of the time-varying model, which can be calculated by the Kalman filter algorithm, and η(t) represents the multivariate independent white noise. The symbol p denotes the MVAAR model order selected by Schwarz Bayesian Criterion (Schwarz, 1978; Wilke et al., 2008; Tian et al., 2018b).

After obtaining the coefficients of the MVAAR model, we calculated the ADTF by applying Equation (5) to convert the model coefficient ω(j,t) to the frequency domain. The $H_{ij}$ element of H(f,t) describes the directional information flow between the *j*th and the *i*th element at each time point t as:


(5) ω(f,t)∗X(f,t)=η(f,t)

(6) $$X\left(f,t\right)=\omega^{-1}\left(f,t\right)*\eta \left(f,t\right)=H\left(f,t\right)*\eta \left(f,t\right)$$

where $\omega \left(\mathrm{f,t}\right)=\overset{p}{\underset{k=0}{\sum }}\omega_{k}\left(t\right)e^{-j2\pi f\cdot \mathrm{tk}}\omega_{k}$ is the matrix of the time-varying coefficients. ω(f,t) and η(f,t) are transformed into the frequency domain as X(t) and η(f,t), respectively.

Defining the directed causal interrelation from the *j*th to the *i*th element, the normalized ADTF is described between (0,1) as follows:


(7) $$\iota_{\mathrm{ij}}^{2}\left(\mathrm{f,t}\right)=\frac{{\left|H_{\mathrm{ij}}\left(\mathrm{f,t}\right)\right|}^{2}}{\sum_{k}^{n}{\left|H_{\mathrm{ik}}\left(\mathrm{f,t}\right)\right|}^{2}}\;$$

To obtain total information flow from a single node, the integrated ADTF is calculated as the ratio of summed ADTF values divided by the interested frequency bands (*f*_1_, *f*_2_):


(8) $$\upsilon_{\mathrm{ij}}^{2}\left(t\right)=\frac{\sum_{f1}^{f2}\iota_{\mathrm{ij}}^{2}\left(\mathrm{k,t}\right)}{f2-f1}$$

Surrogate data were used to establish the empirical ADTF value distribution under the connectionless zero assumption since the ADTF function has a highly non-linear correlation with the time series it derives, making it impossible to determine the distribution of the ADTF estimator under zero assumption without causality. The shuffling procedure independently and randomly iterated Fourier coefficient phases to produce new surrogate data while preserving the spectral structure of the time series (Wilke et al., 2008). To establish a statistical network, the nonparametric signed rank test was used to select statistically significant edges. The shuffling procedure was repeated 200 times for each model-derived time series from each participant to obtain the significance threshold of *p* < 0.05 with Bonferroni correction (Tian et al., 2018b).

### 2.10. Correlation analysis

The relationship between the information flow and the corresponding average response time (RT) was calculated using Pearson correlation based on the results of time-varying network analysis.

## 3. Results

### 3.1. Behavioral data analysis

Significant effects were observed for SOA (*F*[1,14] = 9.85, *p* < 0.01) and validity (*F*[1,14] = 8.74, *p* < 0.05), as well as their interaction (*F*[1,14] = 27.54, *p* < 0.001). However, no significant visual field effect (*F*[1,14] = 3.60, *p* > 0.05) or interactions between visual field and SOA or validity were found.

**Figure 3:**
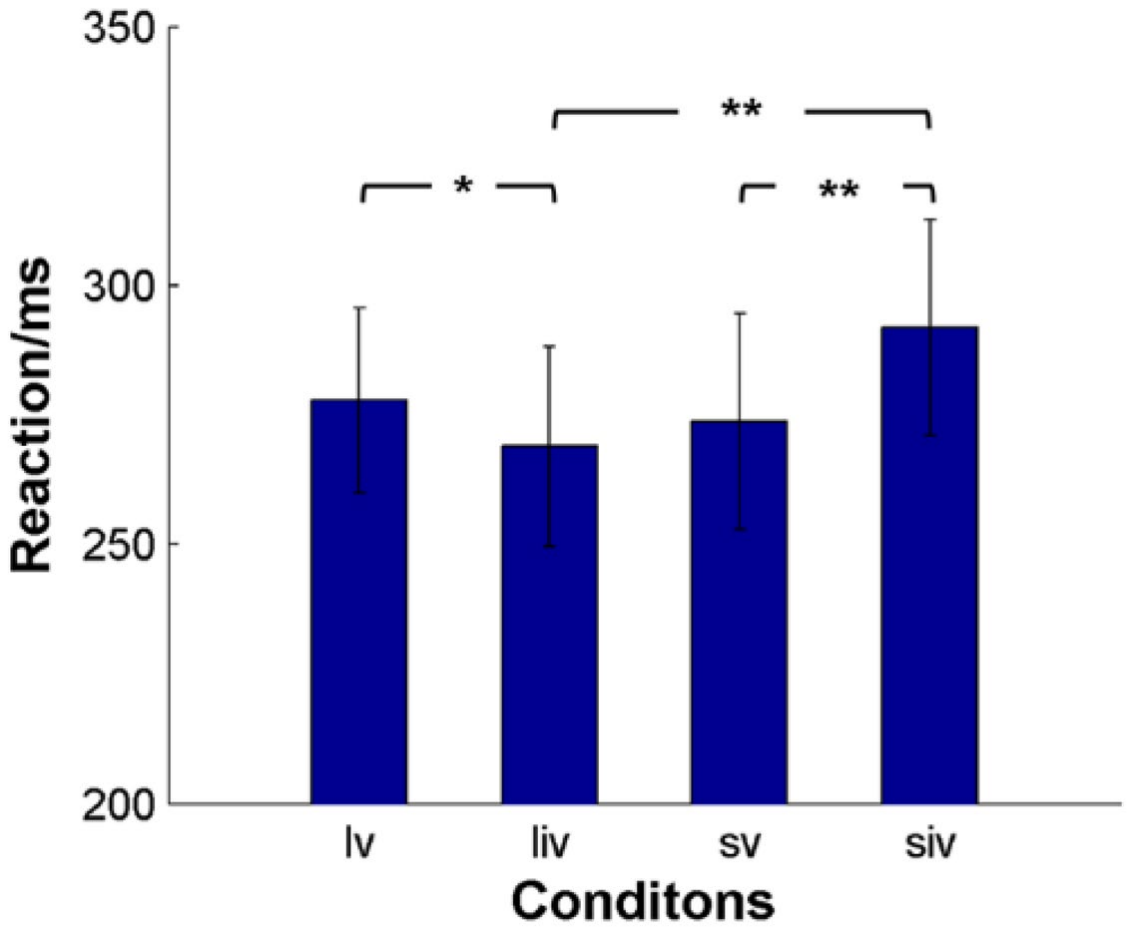
The average response time (RT) of subjects for the four conditions. lv denotes long SOA-valid condition, liv denotes long SOA-invalid condition, sv denotes short SOA-valid condition, siv denotes short SOA-valid condition. **p < 0.001, *p < 0.05.

Because SOA, validity, and their interaction were significant, we conducted paired t-tests for the effects of SOA and validity (Figure 3). The results showed that participants reacted significantly faster in long SOA-invalid trials (268.94 ± 19.33 ms) than in long SOA-valid trials (277.79 ± 17.91 ms). In short SOA-invalid trials (291.91 ± 20.76 ms), participants took significantly more time to react than in short SOA-valid trials (273.80 ± 20.87 ms). There were also significant differences between long and short SOA-invalid trials. Although the RTs of long SOA-valid trials were slower than those of short SOA-valid trials, the difference was not significant.

### 3.2. fMRI results

**Figure 4:**
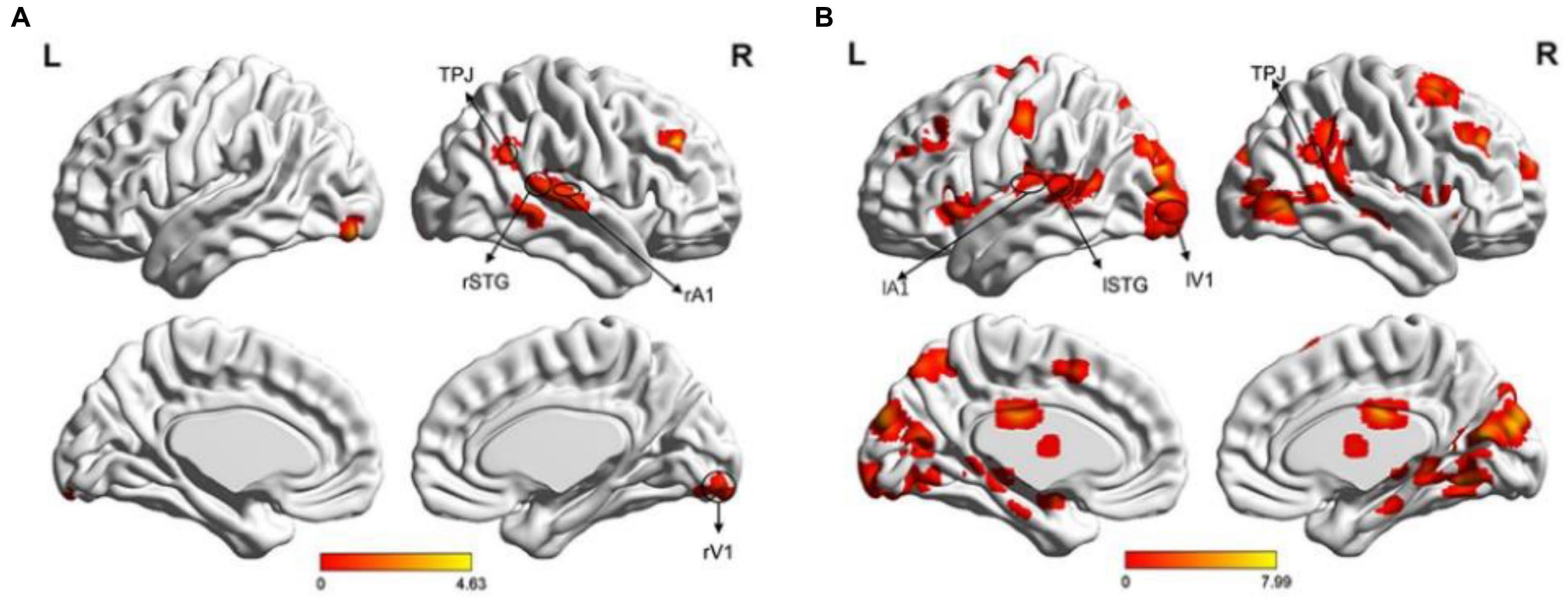
Brain activation maps. **(A)** Illustration of the activation map when the cue stimulus appeared in the LVF. The main activated areas (*p* < 0.05, FDR) included the right TPJ, right STG, right A1, right V1, and right MFG; **(B)** Illustration of the activation map when the cue stimulus appeared in the RVF (*p* < 0.05, FDR correction). The activated areas included the left STG, left A1, left V1, bilateral MFG, right TPJ, and right MT.

A single sample t-test was performed to analyze fMRI data, revealing areas related to visual (V1), auditory (A1), multisensory integration (STG), and attention (angular, middle frontal cortex [MFG]) in both the left and right visual field (LVF and RVF). In the LVF, the main activated areas (*p* < 0.05, FDR correction) included the right angular gyrus (BA39), which is part of the right temporoparietal junction (rTPJ), right STG (BA21), right Heschl’s gyrus as A1 (BA48), right lingual gyrus as rV1 (BA18), and right MFG (BA46), as shown in Figure 4A. In the RVF, more activated areas (*p* < 0.05, FDR correction) included left STG (BA21), left A1 (BA48), left V1 (BA17), bilateral MFG (BAs44/45/46), right TPJ (BA39), and so on, as shown in Figure 4B.

We selected four ROIs based on the task-related fMRI activations depicted in Figure 4 for both LVF and RVF cues. Specifically, when the cue appeared in either the LVF or RVF, the right TPJ (rTPJ) was selected for both LVF and RVF cues. When the cue appeared in the LVF, we chose the right STG (rSTG), right A1 (rA1), and right V1 (rV1). When the cue appeared in the RVF, we chose the contralateral STG, A1, and V1. The coordinates and sizes of the ROIs are presented in Table 1.

**Table 1:** The four selected regions of interest (ROIs) in each visual field.

ROI	MNI coordinates (mm)			The size of ROI (mm)
	*x*	*y*	*z*	
Cue in the LVF				
rTPJ	45	−54	30	6
rSTG	63	−39	18	6
rA1	45	−21	12	10
rV1	9	96	−3	10
Cue in the RVF				
rTPJ	45	−54	33	6
lSTG	−64	−46	18	6
lA1	−40	−26	14	10
lV1	−9	−96	−9	10

### 3.3. Time-varying network

**Figure 5:**
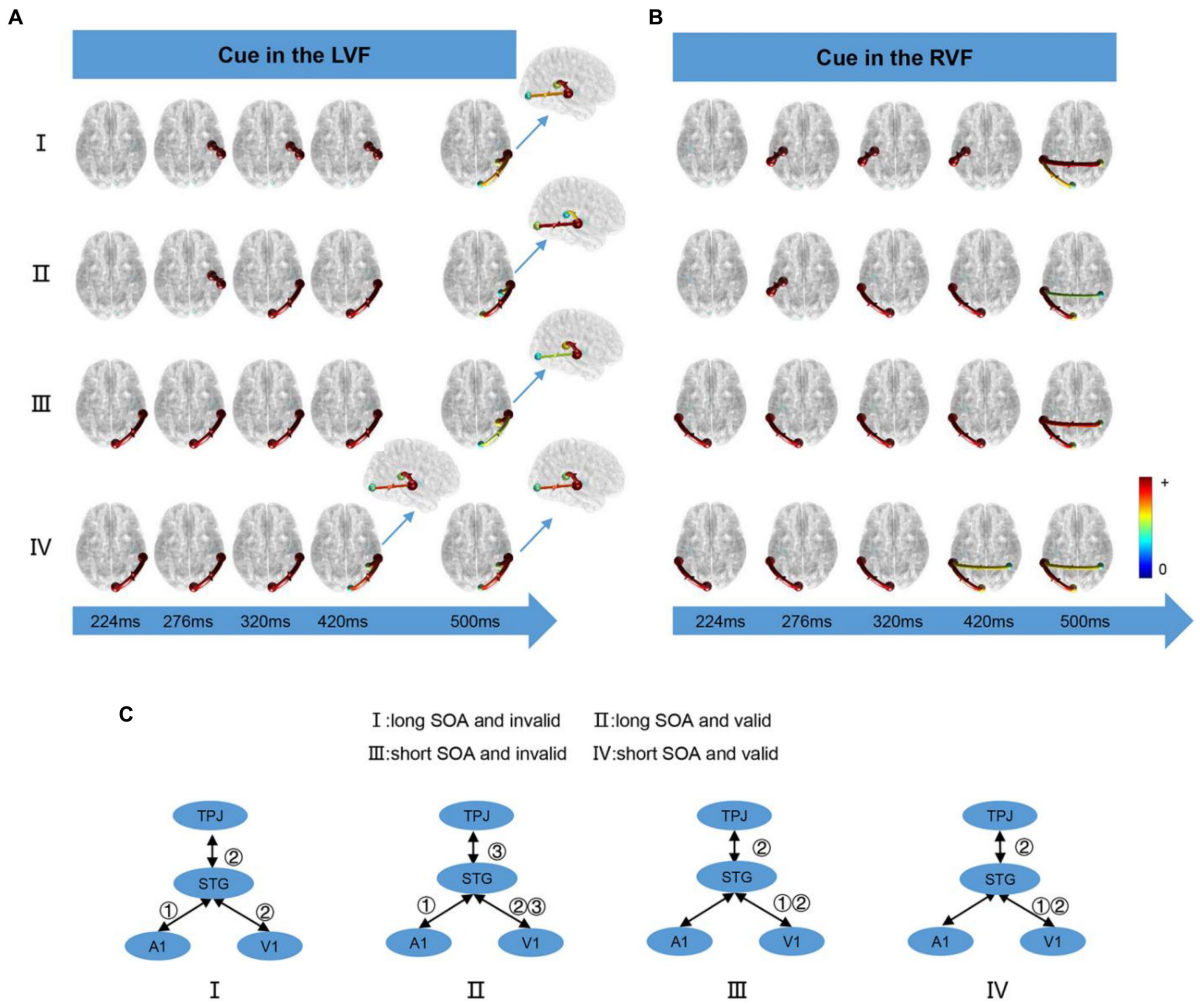
The time-varying networks when the cue stimulus appeared in the **(A)** LVF and **(B)** RVF. **(C)** Summary of time-varying networks. ①②③ denote the order in which the connections appear.

We computed the time-varying network at time points ranging from 200 ms to 900 ms and displayed the connection time points only when it changed in the four conditions. When the cue appeared in the LVF or RVF, the changes in cue conditions were illustrated in Figure 5A and Figure 5B, respectively. Figure 5C summarizes the results of the time-varying network analysis. The first step for the long SOA condition was A1↔STG, whereas for the short SOA condition, it was V1↔STG. The last step for both long and short SOA conditions was V1↔STG and STG↔TPJ. Notably, in the long SOA-valid condition, V1↔STG was the middle step.

### 3.4. Correlation analysis

**Figure 6:**
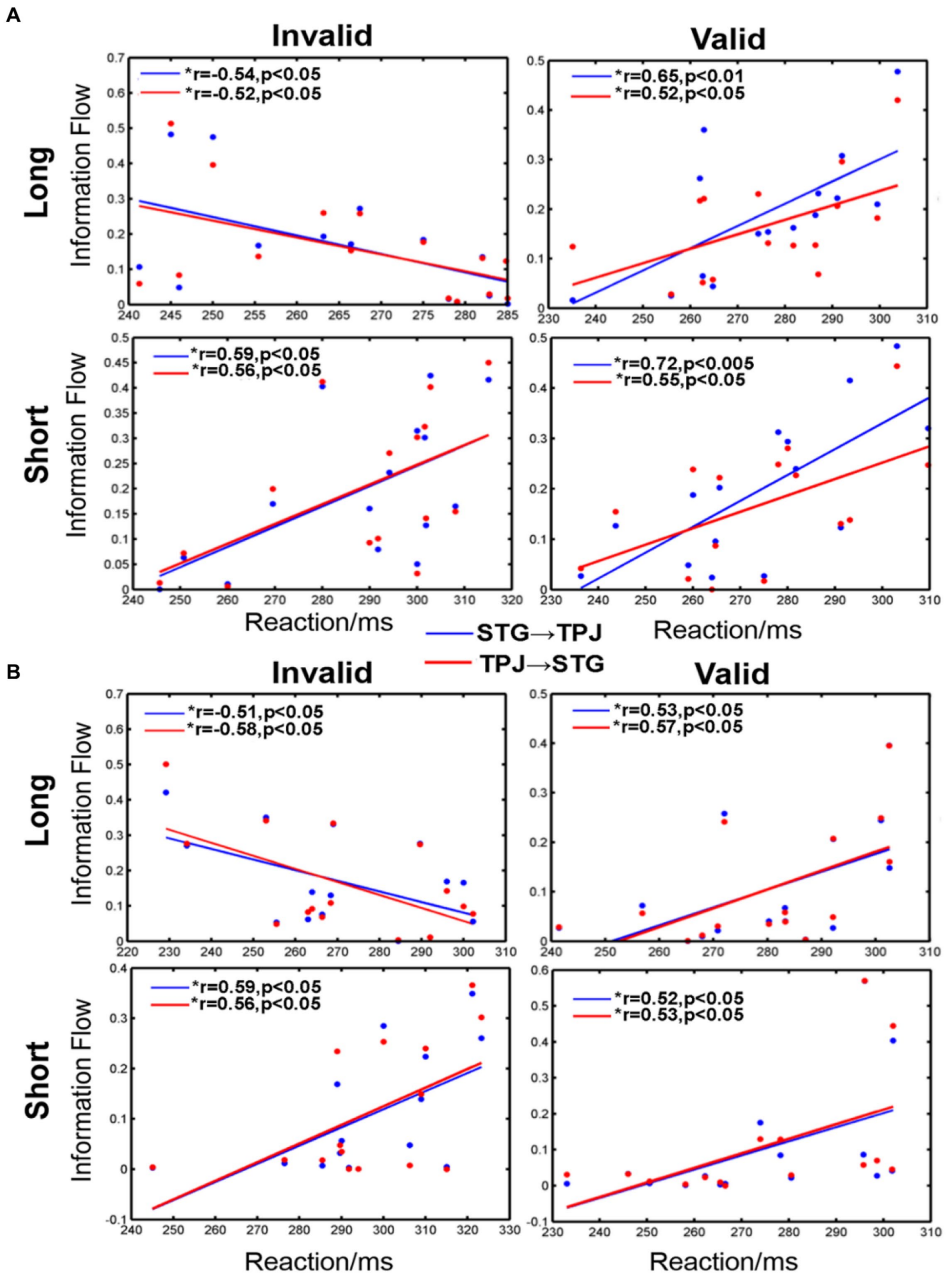
The correlation between information flow and average reaction time (RT) when the cue stimulus appeared in the **(A)** LVF and **(B)** RVF. *a significant difference between the two regions. STG → TPJ denotes the causal flow from STG to TPJ. TPJ → STG denotes the causal flow from TPJ to STG.

Our analysis revealed significant correlations between reaction and information flow (such as STG → TPJ and TPJ → STG) for all conditions, as shown in Figure 6. For the long SOA, distinct differences were observed for each condition. Negative correlations were evident when the cue was invalid and appeared in the LVF (STG → TPJ: *r* = −0.54, *p* < 0.05; TPJ → STG: *r* = −0.52, *p* < 0.05) or RVF (STG → TPJ: *r* = −0.51, *p* < 0.05; TPJ → STG: *r* = −0.58, *p* < 0.05). Conversely, positive correlations were evident when the cue was valid and appeared in either the LVF (STG → TPJ:r = 0.65, *p* < 0.01; TPJ → STG: r = 0.52, p < 0.05) or RVF (STG → TPJ:r = 0.53, p < 0.05; TPJ → STG: r = 0.57, p < 0.05). Similar trends were noted for all conditions for the short SOA, as shown in Figure 6. Positive correlations between mean RT and information flow were observed when the cue was invalid and appeared in the LVF (STG → TPJ: *r* = 0.59, *p* < 0.05; TPJ → STG: *r* = 0.56, *p* < 0.05) or RVF (STG → TPJ: *r* = 0.59, *p* < 0.05; TPJ → STG: *r* = 0.56, *p* < 0.05). Similarly, positive correlations were observed when the cue was valid, regardless of whether it appeared in the LVF (STG → TPJ:r = 0.72, *p* < 0.005 TPJ → STG: r = 0.55, p < 0.05) or RVF (STG → TPJ: r = 0.52, p < 0.05; TPJ → STG: r = 0.53, p < 0.05)

## 4. Discussions

In this study, the behavioral results showed that the RT for a valid cue was significantly shorter than an invalid cue in the short SOA condition, while the opposite was opposite for the long SOA, which was similar to the unimodal task. In both long and short SOA conditions, we observed STG activation, a critical auditory–visual integration region (Klemen and Chambers, 2012). Additionally, we observed activation in TPJ and MFG, which are important VAN areas (Corbetta et al., 2008), indicating that attention plays a role in auditory–visual integration. Our time-varying network analysis revealed that V1/A1↔STG occurred before TPJ↔STG, as shown in Figure 5, indicating that pre-attention in auditory–visual integration.

### 4.1. Similar results observed between the bimodal and unimodal cue-target paradigms

Previous researches have reported that there is a significant cue effect for short SOAs in the visual cue-target paradigm. On the condition of the time interval of the cue and target stimulus is shorter than 300 ms, the subjects exhibited faster responses when the cue was valid as compared to when it was invalid. However, the subjects showed slower responses when the cue was valid rather than invalid for long SOA (more than 300 ms). These findings were consistent with previous studies (Lepsien and Pollmann, 2002; Mayer et al., 2004a,b; Tian and Yao, 2008; Tian et al., 2011) and suggested that stimulus-driven attention effects are faster and more transient than goal-directed attention effects (Jonides and Irwin, 1981; Shepherd and Müller, 1989; Corbetta et al., 2002; Busse et al., 2008; Macaluso et al., 2016; Tang et al., 2016). Similar outcomes have been observed in the auditory paradigm (Alho et al., 2015; Hanlon et al., 2017). Our behavioral analysis aligns with previous research on the unimodal paradigm and suggests that there is no difference between unimodal and bimodal paradigms in the cue-target paradigm.

### 4.2. Integration and attention exist in the bimodal cue-target paradigm

Previous studies have emphasized that auditory–visual integration in the cue-target paradigm occurs when the cue with one modal stimulus and the target with a different modal stimulus are presented from around the same spatial position (Stein and Meredith, 1990; Spence, 2013; Wu et al., 2020) and at approximately the same time (Stein and Meredith, 1990; Frassinetti et al., 2002; Bolognini et al., 2005; Spence and Santangelo, 2010; Stevenson et al., 2012; Tang et al., 2016). However, it will not appear if the cue precedes the target by more than 300 ms (Spence, 2010). In our paradigm, the time intervals between cue and target stimulus were divided into 100 and 800 ms, which cannot be directly compared to previous studies. Our fMRI results, where the STG appeared in all conditions, indicate that auditory and visual integration occurs even when these two stimuli are not aligned in space or time (i.e., more than 300 ms interval).

The role of attention in multisensory integration is still under debate. Some studies proposed that multisensory integration is an automatic process (Vroomen et al., 2001; José et al., 2020), while others suggested that attention played an important role in multisensory integration (Talsma et al., 2007, 2010; Fairhall and Macaluso, 2009; Tang et al., 2016). Since rTPJ and MFG important parts of the ventral attention network (Corbetta et al., 2008; Klein et al., 2021), the experimental activation of these rTPJ and MFG suggested that attention may also be involved in integration. We used time-varying networks to determine the temporal order between multisensory integration and attention using combination of fMRI and EEG data, which allowed for greater precision than EEG data alone. Additionally, the fMRI data provided a more precise spatial resolution for the time-varying networks.

### 4.3. Auditory–visual integration prior to attention

In this research, we constructed a time-varying network using task-related fMRI activations as nodes, including TPJ as the core of the VAN (Corbetta et al., 2008), and STG as an important area for integration (Yan et al., 2015). Our aim was to investigate the relationship between multisensory integration and attention. As depicted in Figure 5C, the V1/A1↔STG connection was always the first order, followed by STG↔TPJ, regardless of the conditions. This finding supports the notion that pre-attention is involved in auditory and visual integration, which is consistent with previous studies (Erik et al., 2008). However, we observed some differences under different conditions, such as the SOA length. For short SOA, the first connection was V1↔STG, as visual stimuli are dominant in processing spatial characteristics, while auditory events dominate temporal characteristic processing (Bertelson et al., 2000; Stekelenburg et al., 2004; Bonath et al., 2007; Navarra et al., 2010). Conversely, for long SOA, information flowed from the A1 to STG. This result might be due to the auditory–visual stimuli being temporally unsynchronized in our data collection. As the SOA increased, the dominant role of the visual stimulus diminished, and the auditory effect became stronger, leading to a significant A1↔STG flow. Interestingly, TPJ↔STG and STG↔V1 were the last step in all conditions, indicating that the TPJ modulates the primary cortex by using integration areas as a transfer node in all cases.

### 4.4. Relationship between information flow and RT

Numerous studies have investigated how attention affects a subject’s reaction time, but there is disagreement on whether attention boosts or limits the reflection (Senkowski et al., 2005; Karns and Knight, 2009; Macaluso et al., 2016). Some studies have suggested that attention accelerates reaction speed (Mcdonald et al., 2005, 2009; Van der Stoep et al., 2017), while others have proposed that attention may actually inhibit reaction (Tian and Yao, 2008). A recent study has demonstrated that both stimulus-driven attention and multisensory integration can accelerate responses (Van der Stoep et al., 2017; Motomura and Amimoto, 2022).

In this study, we compared the correlation between mean RT and the information flow of STG↔TPJ under different circumstances. Our findings suggest that attention has a direct influence on multisensory integration, as the extent of information flow reflects the mutual influence of the two brain regions. Specifically, we observed a negative correlation between the two regions in the long SOA-invalid condition, indicating that larger information flow led to faster reflection times. We inferred that this phenomenon is due to bottom-up attention, where increased information flow leads to greater information exchange between the STG and TPJ and, thus, faster reactions. However, in other conditions, we observed positive correlations, which we attribute to the modulation of attention. Specifically, greater attention modulation results in inhibited reactions.

## 5. Conclusion

In this paper, our analysis of the behavioral data showed no discernible difference between the multisensory and unisensory cue-target paradigms. We also employed fMRI data analysis to demonstrate the existence of auditory–visual integration in the long SOA condition and the necessity of attention for such integration. The constructed time-varying networks based on fMRI coordinates revealed that multisensory integration occurs prior to attention and pre-attention is involved in auditory–visual integration. Furthermore, our findings suggest that attention can impact the subject’s reaction time, but the effect depends on the situation, and greater attention modulation results in inhibited reactions.

## Data availability statement

The raw data supporting the conclusions of this article will be made available by the authors, without undue reservation.

## Ethics statement

The studies involving human participants were reviewed and approved by the Ethics Committee of the University of Electronic Science and Technology of China. The patients/participants provided their written informed consent to participate in this study.

## Author contributions

YJ performed experiments and data analysis. RQ and YS contributed to data analysis. YTa wrote the draft of the manuscript. ZH and YTi contributed to experiments design and conception. All the authors edited the manuscript. All authors contributed to the article and approved the submitted version.

## Funding

This work was sponsored in part by the National Natural Science Foundation of China (Grant No. 62171074), China Postdoctoral Science Foundation (No. 2021MD703941), special support for Chongqing postdoctoral research project (2021XM2051), Natural Science Foundation of Chongqing (cstc2019jcyj-msxmX0275), Project of Central Nervous System Drug Key Laboratory of Sichuan Province (200028-01SZ), the Doctoral Foundation of Chongqing University of Posts and Telecommunications (A2022-11), and in part by Postdoctoral Science Foundation of Chongqing (cstc2021jcyj-bshX0181), in part by Chongqing Municipal Education Commission (21SKGH068).

## Conflict of interest

The authors declare that the research was conducted in the absence of any commercial or financial relationships that could be construed as a potential conflict of interest.

## Publisher’s note

All claims expressed in this article are solely those of the authors and do not necessarily represent those of their affiliated organizations, or those of the publisher, the editors and the reviewers. Any product that may be evaluated in this article, or claim that may be made by its manufacturer, is not guaranteed or endorsed by the publisher.
